# Glycan-based scaffolds and nanoparticles as drug delivery system in cancer therapy

**DOI:** 10.3389/fimmu.2024.1395187

**Published:** 2024-05-10

**Authors:** Henan Qin, Yibin Teng, Rui Dai, Aman Wang, Jiwei Liu

**Affiliations:** ^1^ Department of Oncology, The First Affiliated Hospital of Dalian Medical University, Dalian, China; ^2^ Department of Pharmacy, Peking Union Medical University Hospital, Beijing, China

**Keywords:** glycan, scaffolds, cancer, drug, glycosylation

## Abstract

Glycan-based scaffolds are unique in their high specificity, versatility, low immunogenicity, and ability to mimic natural carbohydrates, making them attractive candidates for use in cancer treatment. These scaffolds are made up of glycans, which are biopolymers with well biocompatibility in the human body that can be used for drug delivery. The versatility of glycan-based scaffolds allows for the modulation of drug activity and targeted delivery to specific cells or tissues, which increases the potency of drugs and reduces side effects. Despite their promise, there are still technical challenges in the design and production of glycan-based scaffolds, as well as limitations in their therapeutic efficacy and specificity.

## 1 Introduction

**Figure 1:**
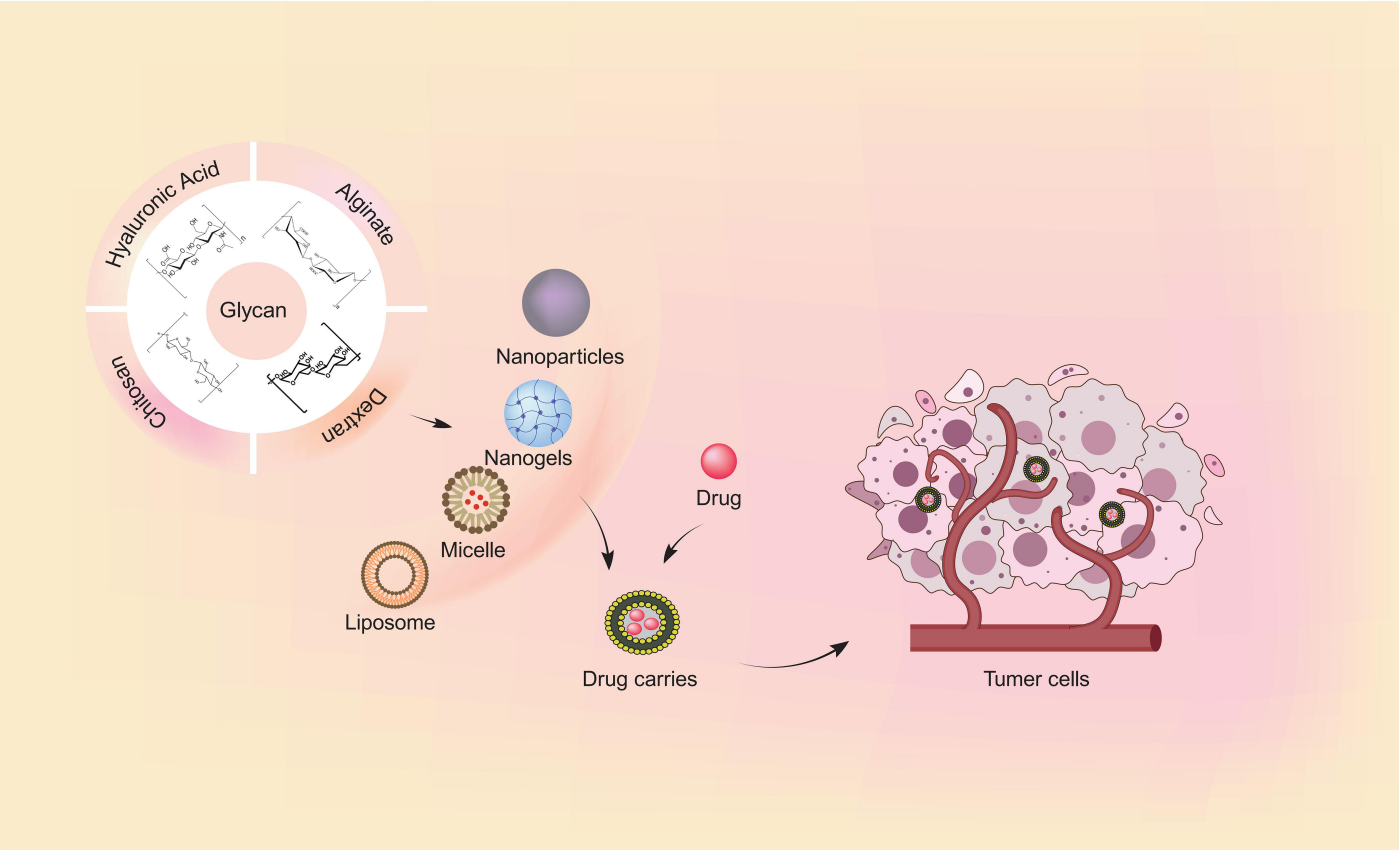
The common forms of glycan-based scaffolds and targeted delivery of drugs. Natural polymers utilized in drug delivery systems include chitosan, hyaluronic acid, dextran, and alginate. The uses of natural polymers to construct nanobiocomposites with anti-cancer agents is advantageous for targeted drug delivery and improving the antitumor efficacy.

Natural polysaccharides contain a large number of oxygen atoms, light-weighted radical groups, and glycosyl groups, enabling them to interact with each other through dipole-dipole, ion-dipole, and hydrogen bonding interactions with themselves or other substances in the solution (1–3). From a molecular structure perspective, polysaccharides are high-molecular-weight carbon-hydrate compounds formed by the condensation of monosaccharides through glycosidic bonds (4, 5). The diversity of polysaccharides in terms of structure and performance is due to differences in the structures of the minimum repeating units (types of monosaccharides), the condensation sites (positions of glycosidic bonds), the repeating orders, and the straight/branched chains and final molecular weights (6, 7). Natural polysaccharide molecules present a three-dimensional regular conformation, with a helical structure in the solid state. This structure exists in the solution under given thermodynamic conditions and is typically subject to conformational transitions when temperature and counterion concentration increase. Natural polysaccharides can be divided into random coil, hyperbranched sphere, and triple helix in solution based on different chain conformations. Natural polysaccharides exhibit good safety, biocompatibility, and biodegradability and are inexpensive and readily available. Additionally, some of them show structural similarity with human extracellular matrix components. Given this background, natural polysaccharides have unparalleled advantages as the carrier material for drug-targeted controlled release systems in scaffold applications. The diversity and richness of natural polysaccharides mean that they can provide a range of biocompatible and biodegradable delivery systems with biological and chemical functions for drugs (**Figure 1**) (8, 9).

Cancer, alongside cardiovascular disease, is one of the most widespread and fatal illnesses worldwide (10). Various cancer drug treatments are available, including chemotherapy, immunotherapy, and targeted therapy (11). However, many patients discontinue treatment due to the side effects, which reduce the efficacy of the treatment, affect their quality of life, and shorten their survival time. Therefore, it is imperative to create a new type of drug that can specifically target cancer cells without harming normal cells. The Glycan-Based Scaffolds drug delivery system has emerged as a crucial component in cancer treatment in recent years (12). Several glycan polymers such as chitosan (CS), hyaluronic acid (HA), dextran (DEX), alginate (ALG), and O-glycosylation can be chemically modified to selectively target the affected area and deliver the drug in a controlled manner. This results in a safe and effective platform for drug delivery that minimizes side effects and enhances the therapeutic efficacy of cancer drugs.

Glycans have emerged as valuable multifunctional drug delivery carriers with significant promise in tumor cell-targeted drug delivery systems. Numerous experimental methodologies have been devised for co-delivery of drugs via nanocarriers. Specifically, in breast cancer, gemcitabine-Trimethyl CS NPs not only enhanced oral bioavailability but also exhibited a notable reduction in tumor growth rate (13). CS/ALG NPs loaded with curcumin diglutaric acid exhibited enhanced stability in simulated gastrointestinal conditions. *In vitro* studies revealed superior inhibitory effects on viability, increased cellular uptake, and heightened cytotoxicity against human epithelial colorectal adenocarcinoma (14). Addressing chemoresistance, particularly challenging in pancreatic cancer treatment, polymer NPs offer a potential solution by facilitating the delivery of chemotherapy drugs to pancreatic cancer cells, thereby circumventing natural barriers (15, 16). In the clinical management of melanoma, persistent challenges include overcoming skin physiological barriers and combating resistance, which significantly undermine treatment effectiveness (17, 18). A recent study employed electrostatic binding to encapsulate DOX within CS/ALG NPs, leading to enhanced DOX accumulation and prolonged cytotoxic effects in melanoma cell lines (19). Furthermore, these glycans have demonstrated attributes such as biodegradability, biocompatibility, low toxicity, and the ability to target cancer cells, thereby augmenting drug efficacy. In this review, we will review the current research on the application of Glycan-Based Scaffolds in cancer therapy.

## 2 Glycan based materials

Glycans are carbohydrate compounds composed of polyhydroxy aldehydes or ketones that can exist in their free form as monosaccharide units or can be covalently linked through glycosidic bonds to form oligosaccharides and polysaccharides (20–22). These carbohydrate molecules can attach to other molecules, such as proteins and lipids, forming glycoconjugates (23). Moreover, glycans can form complexes with other molecules and undergo various modifications, including sulfation, phosphorylation, and acetylation (24). The term glycoconjugate refers to any carbohydrate or assembly of carbohydrates covalently attached to another molecule (25). These free polysaccharides or mono-, oligo-, or polysaccharides can conjugate with a diverse range of biological molecules, including glycoproteins, glycolipids, and peptidoglycans.

### 2.1 Glycosylation

Glycosylation is a tightly controlled enzymatic process involving the attachment of an initial monosaccharide to a non-glycosylated aglycone, through the action of glycosyltransferases and glycosidases (26). The aberrant expression and subcellular localization of glycosyltransferases and glycosidases can contribute to the emergence of abnormal glycosylation, which is predominantly expressed on the cell membrane of tumor cells (27). Targeted drug delivery strategies often rely on the recognition of specific cell surface molecules. Therefore, an ideal target antigen should be widely expressed on the membrane of tumor cells, while being absent or minimally expressed on healthy cells. The expression of aberrantly glycosylated forms of cell surface glycoconjugates in cancer cells presents an attractive target for highly selective and specific drug delivery systems, due to the potential for increased specificity and selectivity conferred by the unique glycosylation patterns of these molecules (28, 29).

The O-glycosylation is a covalent post-translational modification in which monosaccharides are transferred to serine and/or threonine residues of specific proteins by an O-glycosidic bond (30). N-glycosylation is initiated by the synthesis of a dolichol lipid-linked oligosaccharide precursor, which is transferred en bloc to the asparagine residues of nascent proteins (31). Biosynthetic studies showed that the HA-binding function was acquired with N-glycan structures, and in the presence of tunicamycin, with the O-glycan structures, suggesting a positive role for both N- and O-linked oligosaccharides (32).

### 2.2 Chitosan

There has been a great deal of interest in CS, which is a natural biopolymer that is primarily obtained from the shells of crustaceans (33). As a semi-crystalline polymer, CS is derived from chitin, the main component of exoskeletons of crustaceans, by removing the acetyl groups. This cationic polymer is a linear polysaccharide composed of repeating units of β-(1-4) linked d-glucosamine and N-acetyl-d-glucosamine (33). This cationic character allows CS to form complexes with polyanions (34). Furthermore, the exceptional attributes of CS, including its limited solubility in water and acids, biodegradability, biocompatibility, non-toxicity, antibacterial properties and anti-adhesion characteristics, have garnered significant attention (35, 36). This unique chemical structure and properties give CS its unique properties, making it a highly sought-after material in various fields, such as biomedicine. In particular, CS has gained significant attention as a carrier for targeted drug delivery, as it enables sustained drug effects at the subcellular level, resulting in highly accurate cellular targeting and enhanced therapeutic efficacy while minimizing adverse effects (37).

Nanoscale CS (nano-CS) offers unique advantages over conventional CS due to its enhanced permeability, improved biocompatibility, elevated charge density, and superior support for cellular growth. The functional groups on CS nanoparticles (NPs), such as amino or hydroxyl groups, can be readily modified to achieve more precise drug release (38). CS has three vital functional groups consisting of an amino group (NH2 at C-2), abundant primary hydroxyl groups (OH at C-6), and secondary hydroxyl groups (OH at C-3) (39). These functional groups can easily generate intermolecular hydrogen bonds without disturbing their polymerization and allow modification of CS chain copolymerization crosslinked with other polymeric chains which can enable the fabrication of a wide range of composite scaffolds (33). CS scaffolds can be functionalized with various molecules such as drugs, growth factors, or peptides to enhance their therapeutic effects. Moreover, nano-CS can mediate drug and nucleic acid therapeutic delivery in cancer therapy (40, 41). The fabrication of CS derivative nanoparticles typically involves a combination of methodologies such as ionotropic gelation, polyelectrolyte complex formation-induced gelation, polymer-drug complexes formation, and self-assembly (42). CS improves the stability, bioavailability, and controlled release of unstable drugs by leveraging its nanoscale properties (43). The deliberate design of CS-based nanocarriers is aimed at optimizing drug delivery, with a primary focus on enhancing encapsulation efficiency to improve the pharmacokinetic properties of drugs (44). To attain the desired drug encapsulation outcomes, careful consideration of various factors is crucial during the selection of appropriate drug loading techniques, the water solubility of agents tends to be the paramount consideration (45).

### 2.3 Hyaluronic acid

HA is a long and unbranched polysaccharide which was naturally present in all vertebrate animals and human beings Pro-Inflammatory (46). The weight of HA can vary greatly, ranging from as low as 5 kDa to as high as 20,000 kDa *in vivo (* 47). In terms of structure, the HA structure is a type of non-sulfated glycosaminoglycan that is composed of repeating units of N-acetyl D-glucosamine and D-glucuronic acid, linked together through glycosidic bonds in specific arrangement bonds which are formed between alternating β-(1 - 4) and β-(1- 3) linkages (48, 49). Hyaluronan is synthesized by three distinct isoforms of hyaluronan synthase (HAS 1-3) at the plasma membrane. It is a versatile molecule that can be obtained through various methods, including extraction from animal tissues, microbial production, or enzymatic synthesis (47, 50).

HA has been demonstrated to play a crucial role in cellular biology. As a result of forming a pericellular coating around the majority of cells, HA functions as a signaling molecule interacting with its binding proteins (50). The stability within the bloodstream and the capacity to deliver drugs effectively to target cells, thereby enhancing therapeutic efficacy, have positioned HA as a prominent focus in drug delivery research (51). Due to its desirable attributes, such as chemical versatility, biodegradability, high hydrophilicity, and non-toxicity, HA has been successfully utilized as a nanocarrier for drug delivery in cancer chemotherapy (52, 53). HA has been found to possess the capability to selectively target CD44-expressing fibroblasts, thus providing a foundation for its utilization as a promising vehicle in the evolving field of cancer therapeutics (53).

HA is capable of forming a polymeric crosslinked network that exhibits a high water-absorbing ability (54). Chemical modifications enrich the spectrum of processing and manufacturing techniques that can be used to create 3D HA scaffolds. By easily changing the treatment method, HA hydrogels, granular hydrogels (microgels), and HA-based composites can be formed (55–57). HA nanogels display superior colloidal stability and robust encapsulation capability, achievable through diverse fabrication techniques like polyelectrolyte complexation, self-assembly, and chemical crosslinking (58). HA scaffolds can be an attractive material for drug delivery applications, where they can be used to encapsulate and slowly release therapeutic agents at the site of the tumor. Overall, HA scaffolds have demonstrated great promise as a versatile and biocompatible platform for tissue engineering and regenerative medicine.

### 2.4 Dextran

DEX is one of the natural polymers that was discovered from slime-producing bacteria, and subsequent studies have shown that DEX can be produced by several gram-positive, facultatively anaerobe cocci such as Leuconostoc and Streptococcus strains (59). In terms of its molecular structure, DEX is composed of a monomeric unit of α-D-glucose, with a backbone containing α-(1-6) glycosidic linkages (60). DEX molecules can contain not only α-1,6 glycosidic bonds but also α-1,3 glycosidic bonds, which give rise to a linear polymer structure through 1,6-glycosidic bonds and contribute to a certain degree of branching via 1,3-glycosidic bonds (61). The structure of DEX is characterized by its high molecular weight and branched, complex structure, which gives it unique physical and chemical properties. DEX has hydroxyl groups and terminal aldehyde groups in its structure, which can be chemically modified to create DEX-based biomaterials for a variety of biomedical applications (62, 63).

Due to DEX’s excellent biocompatibility, low immunogenicity, and negligible toxicity, it has garnered considerable interest for use in developing drug delivery systems for various biomedical applications (60). In recent years, numerous DEX-based delivery systems with tailor properties and geometries have been developed, including self-assembled NPs, micelles, and hydrogels which have the characteristics of large drug loading, easy absorption, convenient administration, stable performance, and are a hot spot in the field of controlled release for drug delivery and tissue engineering (64, 65). Consequently, DEX-based biomaterials have been extensively investigated as promising carriers for the delivery of therapeutic agents.

### 2.5 Alginates

ALG are linear unbranched anionic polysaccharides found in the cell walls of brown algae, which are generally isolated from brown algae such as Laminaria japonica, Laminaria hyperborea, and Laminaria digitata or soil bacteria such as Azobacter vinelandii (65, 66). ALG being a hydrophilic biopolymer offers substantial features that include biodegradability, biostability, biocompatibility, mucoadhesive features, non-toxicity, hydrophilicity, environmentally -benign properties, and cost-effectiveness that are highly desirous for various biomedical and healthcare applications (67). As a pH-sensitive polymer, serves as a thickening and gel-forming agent and plays a vital role in the sustained/controlled release of drug products (68). A critical property of ALG is that, with certain crosslinking divalent cations, sodium ALG solution can undergo a sol-to-gel transformation, thus it is considered an ideal candidate for drug delivery system applications (69, 70).

ALG drug delivery systems, such as nanogels, hydrogels, and NPs, have been studied extensively for cancer therapy because of their targeted manner to the specific areas of cancer and thereby significantly reducing the drug dosage with enhanced bioavailability (68, 71). ALG presence enhances nanosystem stability in acidic biological fluid environments (67). Due to the nanoporous nature of ALG gels, they are ideally suited for the rapid diffusion of small molecules via gel formation (54). Thus, the application of ALG-based hydrogels loaded with drugs, growth factors, and other bioactive molecules has gained considerable interest from many research groups (72). In addition, ALG has received considerable attention to be used as a carrier in polymeric & nanocarriers (55). The addition of cationic polyelectrolytes can stabilize NPs composed of ALG (73). ALG-based nanosystems exhibited controlled drug release, increased stability, enhanced drug-loading capacity, and reduced immunogenicity, which renders them attractive drug delivery for cancer therapy applications (74, 75). Utilizing ALG for anticancer drug encapsulation significantly enhances the efficacy of anticancer drugs against various cancers (67). ALG formulations produced via techniques like ionic gelation, emulsification, spray drying, and freeze drying, facilitate customized drug loading, improved stability, and prolonged release kinetics (76).

## 3 Glycan and glycoconjugates as drug carriers in the management of cancer

### 3.1 Chitosan

#### 3.1.1 PH-sensitive capsules

Oral administration was the most suitable route for the delivery of therapeutics, which reached the target site through systemic circulation (77, 78). The oral targeting system facilitates the therapeutic efficacy of the drug; and at the same time minimizes the toxic or adverse effect, but the drawback of oral administration is that the drug concentration may not be sufficient (79, 80). To retain drug concentration regionally, a pH-sensitive capsule functionalized LbL assembled film for the targeted release of chemotherapy drugs to treat cancer was prepared. 5-fluorouracil (5-FU) is commonly used as adjuvant chemotherapy in colorectal cancer patients. This capsule would remain stable at pH < 7.0 thereby allowing the LbL film to retain 5-FU till it reaches the large intestine which was composed of CS and ALG polyelectrolytes. A folic acid-conjugated CS layer was added for cancer targeting at the same time. The functionalized layer-by-layer (LbL) film had directional 5-FU release and showed greater cytotoxicity toward colon cancer cell lines (78) Capecitabine (CAP) is a prodrug of 5-FU (81). It has a short plasma half-life of <0.85 h, CAP is eliminated rapidly from the body and needs frequent administration (82). Sinha et al. developed a multi-particulate system for colon cancer, using chitosan succinate-sodium ALG (CS-SA) macromolecular complex as a carrier to encapsulate CAP. This observation demonstrates that CS-SA effectively maintains the drug in acidic conditions, thereby protecting CAP release and thereby achieving the goal of inhibiting the proliferation of tumor cells (82). One of the common causes of endocrine therapy resistance is the development of ESR1 mutations. Fulvestrant is a first-generation selective estrogen receptor degrader that has been shown to have activity against ESR1 mutant tumors (83). However, it requires intramuscular injection, which can be inconvenient for patients, and has poor bioavailability, which means that optimal drug dosing may not be achievable (84). Xu et al. developed a drug delivery system by encapsulating fulvestrant in silica nanocapsules (SNC)/CS and incorporating it into LbL films. The SNS/CS LbL films were effective in trapping fulvestrant at a pH of 7.4, and when the pH was lowered to 5.0, the release rate of fulvestrant was significantly increased (85).

In addition to the formation of pH-sensitive capsule film to facilitate the encapsulation of chemotherapy cells, CS employs various other means to improve the absorption efficiency of orally administered chemotherapy drugs.

#### 3.1.2 Drug aqueous solubility

CS can be used to deliver hydrophilic but short half-life and low permeability drugs to enhance anti-tumor activity. Trickler et al. developed CS/glyceryl monooleate (GMO) NPs. The gemcitabine (GEM) was encapsulated in the NPs which exhibited increasing in cellular accumulation, intracellular internalization, and GEM-induced cytotoxicity, leading to improved anti-tumor activity against pancreatic cancer cells *in vitro (* 86).

CS can be also used to modify aqueous solubility to deliver hydrophobic drugs. Curcumin (CUR) is characterized by low aqueous solubility limiting the clinical application for tumor therapy (87, 88). The micelle is composed of glycyrrhetinic acid-modified CS-cystamine-poly (ϵ-caprolactone) copolymer polymer to deliver CUR into hepatoma cells, resulting in rapid release of the CUR (89). The clinical use of paclitaxel (PTX) as an antineoplastic agent also has been limited by its poor solubility (90). An injectable drug delivery depot system was developed for targeted delivery of PTX against ovarian cancer cells. The system is composed of a hydrogel made of glycol CS (GC), and it contains beta-cyclodextrin (β-CD) that has been complexed with PTX (GC/CD/PTX). By utilizing the CD/PTX complex, PTX’s water solubility has been improved, allowing it to be released quickly over seven days and enhancing the effectiveness of PTX against ovarian cancer cells (91). A drug delivery system known as CS/GMO has been also developed to encapsulate PTX. In comparison to the free form of PTX, the CS/GMO formulation containing PTX showed a nearly fourfold increase in efficacy (92).

#### 3.1.3 Drug-resistant

Polyelectrolyte multilayer capsules, assembled using the LbL technique have been used in many applications in medical fields such as cancer treatment (93). The success of cancer chemotherapy is often limited by multidrug resistance (MDR), which has been identified as a significant obstacle to effective treatment (94). Fernando et al. described docetaxel (DOX)-loaded bovine serum albumin (BSA)-gel-capsules with remarkable antitumor activities against drug-resistant breast cancer. DOX-loaded BSA-gel-capsules were used for antitumor studies *in vitro* with a DOX-resistant cell line (MCF-7/ADR cells). The results demonstrated more effective cytotoxicity against MCF-7/ADR cells compared to free DOX. Additionally, prolonged retention in the tumor site and high DOX accumulation were demonstrated, highlighting the unique advantages of BSA-gel capsules for local chemotherapy of drug-resistant breast cancer (95).

#### 3.1.4 Drug-targeted delivery

ADC (Antibody-Drug Conjugate) drugs are a class of biopharmaceuticals that combine monoclonal antibodies with chemical drugs. They achieve targeted therapy of cancer cells by conjugating a monoclonal antibody that specifically binds to the tumor surface with a cytotoxic chemical drug. ADC drugs consist of three main components: a cytotoxic drug, a monoclonal antibody (mAb), and a flexible linker (96). The underlying mechanism of this strategy is based on the highly specific recognition of a cellular surface antigen, whose expression is highly restricted to the cancer cell population, by the mAb moiety. The cytotoxic payload is then delivered to the tumor tissues through the mAb (97). Such systems can improve the efficacy of chemotherapy while simultaneously minimizing the drug’s toxicity and side effects on the systemic level. Nano-CS is used as the subject for targeted delivery of chemotherapeutics to cancer cells, taking advantage of surface receptors that are specifically overexpressed in these cells (98).

Under this mechanism, the uptake of CS-chemotherapy drugs-mAb by Her2-positive cancer cells was markedly enhanced relative to that of non-targeted CS-DOX NPs and free drugs. By covalently conjugating DOX to CS, CS-DOX conjugate nano aggregates were synthesized. Trastuzumab is intended to act as a targeting ligand for delivery of the DOX to Her2-overexpressing cancer cells. Trastuzumab-decorated NPs demonstrated the ability to selectively target and distinguish between Her2-positive and Her2-negative cells, making them a promising option for breast cancer treatment (99). In pancreatic cancer, due to GEM’s short plasma half-life and rapid inactivation by plasmatic enzymes, the therapeutic potential becomes significantly reduced (100, 101). Trastuzumab-conjugated GEM-loaded Nano-CS (Her2-GEM-CS-NPs) exhibited superior antiproliferative activity, leading to apoptosis via an enhanced S-phase arrest. Anti-Her2 conjugated NPs significantly enhanced the overall antiproliferative activity of GEM and exhibited better selectivity for target cells and tissues (102). Some recent examples of CS-based drug delivery and application are described in **Table 1**.

**Table 1:** A summary of Chitosan‐based nanostructures for drug delivery in cancer treatment.

Compound/Drug	Cancer type	Cell line/animal model	Status	Effect	References
5-FU	Colon cancer	Caco-2 and COLO 320DM colorectal cancer cells	*In vitro*	Greater cytotoxicity towards colon cancer cell lines; Target and regional drug delivery to treat colon cancer;	(64)
CAP	Colon cancer	HT-29 cells	*In vitro*	Inhibition of the proliferation of tumor cell; Prolong the release of CAP in the colonic region; Enhance antitumor efficacy;	(66)
GEM	Pancreatic cancer	BxPC-3 and Mia PaCa-2 cell	*In vitro*	Increase in cellular accumulation; Intracellular internalization and gemcitabine induced cytotoxicity;	(72)
DOX and CUR	Liver cancer	HepG2 and HUVEC cells	*In vitro*	Disassemble rapidly; Strong synergistic anti-cancer activity;	(75)
PTX	Ovarian cancer	SKOV3 cell Balb/c nude mice	*In vitro* *In vivo*	Improve the water solubility; Effective PTX delivery in ovarian cancer therapy;	(77)
PTX	Breast cancer	MDA-MB-231 cells	*In vitro*	Lower doses of PTX to achieve a therapeutic effect; Minimize the adverse side effects;	(78)
DOX	Breast cancer	MCF-7/ADR cells Balb/c nude mice	*In vitro* *In vivo*	More effective cytotoxicity; Positive reversal effect on drug-resistance; High DOX accumulation and prolonged retention in tumor site;	(81)
DOX and trastuzumab	Breast cancer	MCF-7 cells	*In vitro*	Discriminate between Her2+ and Her2- cells; Targeted drug delivery and reduction of drug side effects in Her2 breast;	(85)
Trastuzumab and GEM	Pancreatic cancer	Mia Paca 2 and PANC 1 cells	*In vitro*	Better selectivity for target cells; Enhance cytotoxicity;	(88)

### 3.2 Hyaluronic acid

Taking into account the specific binding of HA to receptors on the surface of cancer cells, it can be used as a carrier of other drugs through the formation of conjugates, generating new compounds with promising antitumor effects (47). A series of advantages are expected following the conjugation of HA to cytotoxic agents regarding aqueous solubility, distribution, stability, and efficacy (89, 90). Due to their unique properties, HA-based drug delivery systems have been extensively studied for both passive and active targeting (103).

#### 3.2.1 Nanoparticle strategies

Numerous types of nanostructures have been extensively studied for their ability to deliver cytotoxic molecules, such as DOX, to cancer cells. These nanostructures have been modified with HA to enhance their targeting and uptake by cancer cells, thereby improving their effectiveness as therapeutic agents (104). Zhang et al. developed a DOX-loaded phenylboronic acid functionalized nanogel platform (DOX/PBNG) for efficient delivery of DOX to cancer cells. To further improve the blood-brain barrier permeability, lactoferrin (Lf) was coated onto the surface of nanogels resulting in Lf-DOX/PBNG. Cytological studies revealed that Lf-DOX/PBNG demonstrated enhanced cellular uptake efficiency and significantly higher cytotoxicity towards glioma cells compared to conventional DOX treatment. Additionally, the nanogels showed superior brain permeability, enabling better drug delivery to the brain (105).

#### 3.2.2 Drug-resistant

Resistance to cisplatin is a significant obstacle to effective chemotherapy for non-small cell lung cancer (106). While respiratory administration of the HA-cisplatin conjugate to the lungs may provide therapeutic benefits for the treatment of lung cancer, this approach has the potential to reduce general toxicities and increase deposition and retention of cisplatin in lung tumors, nearby lung tissues, and lymph glands, ultimately leading to improved outcomes and reduced side effects (107).

#### 3.2.3 Drug-targeted delivery

**Figure 2:**
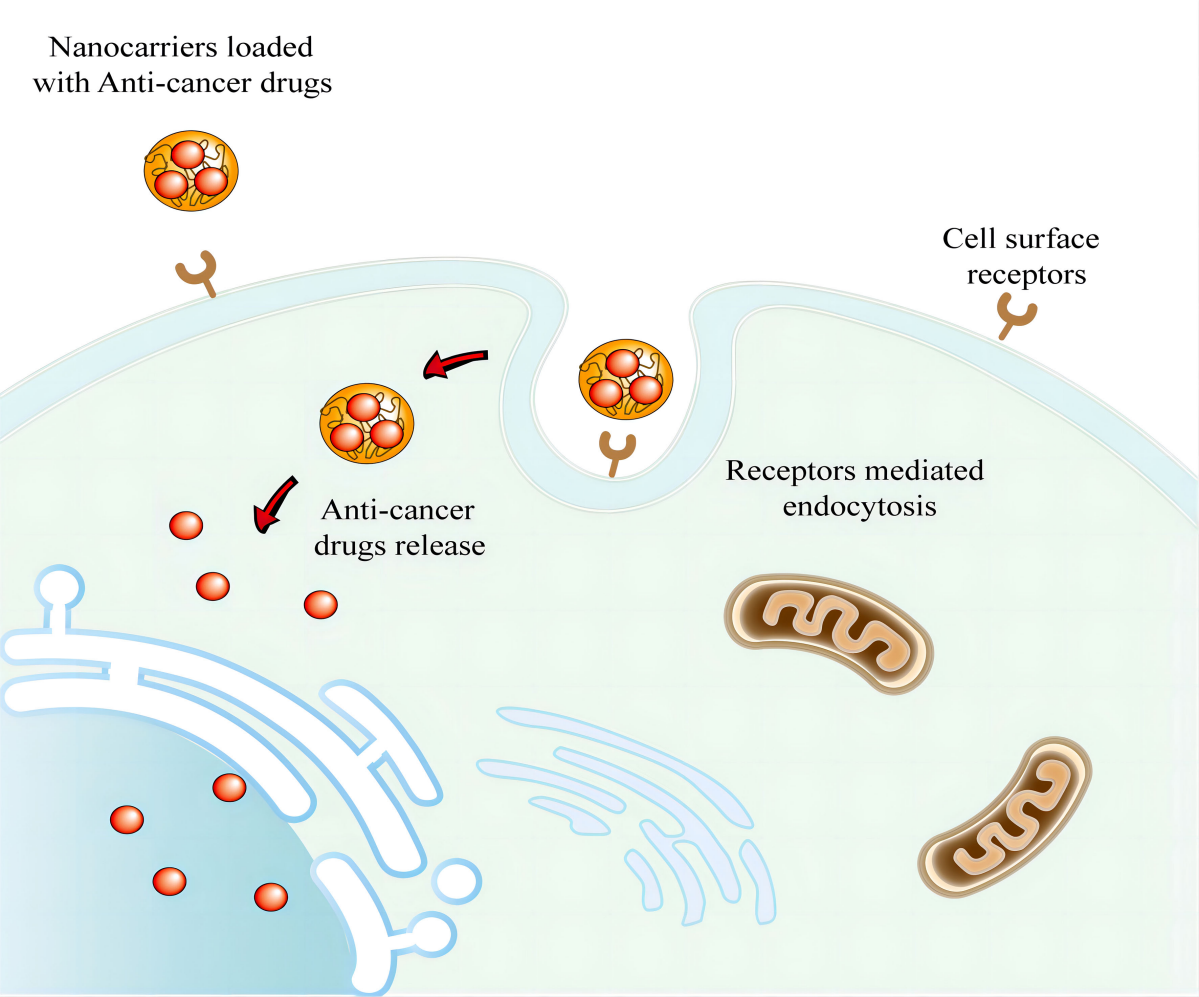
Active targeting via nanocarriers for drug delivery against cancer cells. The ligands of nanocarriers can interact with overexpressed antigens or receptors in tumors. And nanocarriers then enters cancer cells through receptor-mediated endocytosis.

Cellular growth against 4T1 (CD44) is an attractive molecular candidate for targeted drug delivery in cancer treatment (108). Tamoxifen (TMX) is a frequently prescribed selective estrogen receptor modulator for the treatment of breast cancer in patients with hormone receptor-positive tumors (109). However, oral formulations of TMX are known to have high gastric instability and undergo extensive hepatic metabolism, which can result in the need for high doses to achieve therapeutic levels (110). A self-nano-emulsifying drug delivery system (SNEDDS) can improve drug uptake and bypass the first-pass effect in the lymphatic system. Researchers developed a TMX-loaded SNEDDS that targets breast cancer cells overexpressing CD44 receptors. The system utilizes HA-based polymers to increase intracellular uptake 7.11-fold increase compared to pure TMX and targeting while reducing side effects, making it a promising targeted drug delivery system for breast cancer therapy (110). Besides, Tao Yu et al. have developed dual drug-loaded HA micelles by incorporating DOX and cisplatin as chemotherapeutic agents and utilizing HA. The HA-DOX- cisplatin micelles demonstrated significant improvement in drug release under acidic conditions, leading to higher cellular uptake and more effective inhibition of CD44 positive breast cancer cells (111). Furthermore, Wang et al. connected docosahexaenoic acid (DHA) and chlorin e6 (Ce6) to the HA skeleton via Cystamine (cys), resulting in the formation of a redox-sensitive polymer known as HA-cys-DHA/Ce6. NPs were then fabricated and physically encapsulated with DOX. These NPs can specifically bind to CD44 receptors that are highly expressed on the surface of tumor cells, enabling active targeting and offering significant potential for enhancing the efficacy of cancer treatment while reducing toxicity (112). Barbarisi et al. prepared a novel nano hydrogel made of HA that was loaded with quercetin and temozolomide, a commonly used anticancer drug for glioblastoma. The nanocarriers developed in this study were effective in delivering quercetin specifically to glioblastoma cells through the CD44 receptor, thereby improving the therapeutic efficacy of temozolomide (**Figure 2**) (113).

Conventional paclitaxel drugs are bound by cosolvents as well as liposomal phospholayers, resulting in slow-release kinetics, toxicity, and susceptibility to allergic reactions (114). The albumin-bound PTX (Abraxane) as a surfactant-free formulation of PTX has been authorized to mitigate adverse effects (115). However, the clinical utility of Abraxane remains hindered by its high costs and short *in vivo* half-life (116). Due to its capability to target CD44 receptors, which are overexpressed in numerous cancer cells, HA has found extensive use in drug carrier modification (117). A study verified that alkylamine-modified HA enhances the targeting of HA to CD44 and significantly boosts the uptake efficiency of tumor cells (118). Liu et al. modified HA with alkyl amines, subsequently synthesizing amphiphilic HA polymers. These polymers could deliver PTX via self-assembly, resulting in a pronounced inhibition of tumor growth both *in vitro* and *in vivo*.

The above studies demonstrate the potential of drugs conjugated with HA as a novel class of bioconjugated and tumor-targeted chemotherapeutic agents for cancer treatment. Moreover, HA can be integrated into nanomaterials to improve water solubility, biocompatibility, and targetability by specifically binding to CD44-overexpressing cancer cells. Some recent examples of HA-based drug delivery and application are described in **Table 2**.

**Table 2:** A summary of Hyaluronic acid‐based nanostructures for drug delivery in cancer treatment.

Compound/Drug	Cancer type	Cell line/animal model	Status	Effect	References
DOX	Glioma	G422 cells SD rats and ICR mice	*In vitro* *In vivo*	Superior brain permeability; controlled release; Precise glioma dual-targeting;	(93)
Cisplatin	Lung cancer	A549 cells Sprague-Dawley rats	*In vitro* *In vivo*	Higher cisplatin accumulations; relatively non-invasive; more effective;	(95)
TMX	Breast Cancer	MCF-7 cells	*In vitro*	Higher permeation; Safe and compatible against macrophages; Non-toxic nature; Augmented intracellular uptake;	(98)
DOX and cisplatin	Breast Cancer	4T1 cells Balb/c nude mice	*In vitro* *In vivo*	Stronger cellular growth inhibition; Acid-sensitive drug release; CD44-targeted delivery; Excellent biocompatibility and biodegradation;	(99)
DOX	Breast Cancer	MCF-7 cells Balb/c nude mice	*In vitro* *In vivo*	Passive targeting; Effective cellular take; Good biocompatibility better antitumor response;	(100)
Temozolomide and quercetin	glioblastoma	A17 and T98MG cells	*In vitro*	Enhance the specificity and efficacy of temozolomide;	(101)

### 3.3 Dextran

#### 3.3.1 Nanoparticle strategies

Various DEX conjugates, NPs, and micelles are extensively used as nanocarriers in nanomedicine applications. DEX has many advantages as a biopolymer for synthesizing nanomaterials for drug delivery, such as excellent solubility, biocompatibility, biodegradability, and non-immunogenicity (119, 120). Many classes of DEX-based NP systems for anti-cancer drug delivery have been reported in recent years. Park et al. synthesized NPs composed of deoxycholic acid-conjugated DEX (DEXDA), which were loaded with DOX. The *in vitro* cytotoxicity test, conducted using CT26 tumor cells, demonstrated that DEXDA NPs exhibited higher antitumor activity compared to free DOX. Furthermore, the rate of drug release from the DEXDA NPs was found to be accelerated in acidic conditions (121). Another approach for the delivery system is to use a microemulsion method to incorporate the drug into solid lipid nanoparticles (SLNs). Ehsan Aboutaleb et al. explore the optimization and evaluation of cetyl palmitate SLNs complexed with DEX sulfate as a delivery system for vincristine in the treatment of brain tumors. The SLN formulation enhanced the delivery of the drug to the brain close to five times compared to the vincristine solution as well as longer drug mean residence times. It suggested that vincristine-loaded SLNs have great potential as a highly effective drug delivery system for brain tumors (122). Moreover, DEX-CUR NPs were reported to synergize conventional chemotherapeutics such as methotrexate (MTX). Curcio et al. demonstrated that the novel self-assembling DEX-CUR conjugate was able to deliver MTX to MCF-7 cancer cells and increase the therapeutic effects by acting synergistically. The DEX-CUR conjugate NPs were able to effectively deliver MTX to the cancer cells, resulting in enhanced cytotoxic activity and prolonged drug release. The study provides evidence that the DEX-CUR conjugate NPs have the potential to be an effective drug delivery system for the treatment of breast cancer (123). Various DEX-anti-tumor drug conjugates enhance the effectiveness and improve the cytotoxic effects of chemotherapeutic agents (124). As drug delivery carriers, some DEX-based DOX encapsulated and chemically conjugated NPs have shown excellent antitumor activity (125–127). Peng et al. synthesized a polysaccharide DEX-based conjugate for the selective delivery of DOX (128). In the field of oral cancer treatment, nanotechnology also has emerged as an innovative tool that has been shown to overcome the limitations of conventional drug therapies (129). Junichi Nakamura et al. synthesized DEX-Taxol (DEX-TXL) conjugates which formed by linking TXL and its derivatives with aminated DEX which possessed high solubility. Conjugation of folic acid to DEX-TXL resulted in 2-3 times greater anticancer effects in KB cells, which could have important implications for the treatment of oral cavity carcinoma (130).

#### 3.3.2 Micelle

The amphiphilic structure-based DEX polypro-drug, DEX-DOX (DOXDT), has demonstrated the potential to form unimolecular micelles in aqueous media (131). The acidity-sensitive DOXDT prodrug is capable of self-assembling into nanoscale micelles and exhibits excellent micellar stability. The DOXDT delivery system offers significant advantages, including its good water solubility, controlled release in response to acidity, non-toxic nature, and small size of the prodrug micelles. Compared to other lipid-based drug delivery systems, the DOXDT prodrug has a higher drug loading capacity, reaching up to 23.6%. Please note that the text within the parentheses has not been modified (132).

#### 3.3.3 Drug-resistant

To overcome multidrug resistance (MDR), researchers are developing new drugs and treatment strategies. Mitoxantrone (MTO) is a substrate of the efflux transporter breast cancer resistance protein (BCRP), which results in severe resistance to MTO by tumor cells (133, 134). Zhang et al. developed a novel drug delivery system using nanostructured lipid-DEX sulfate hybrid carriers (NLDCs) to overcome multidrug resistance in the treatment of cancer. The MTO-NLDCs could reduce the cardiotoxicity of MTO and enter BCRP-overexpressing MCF-7/MX cells through endocytosis. This mechanism enhances the accumulation of MTO in the resistant MCF-7 cells and overcomes the MDR induced by escaping the efflux induced by the BCRP transporter (135).

Although a tremendous amount of work has been done to develop DEX-based NPs as delivery systems, more *in vivo* studies need to be conducted soon to clarify the changes of NPs in the morphological structure and more clinical toxicity tests need to be carried out before practical application. Some recent examples of DEX-based drug delivery applications are described in **Table 3**.

**Table 3:** A summary of Dextran‐based nanostructures for drug delivery in cancer treatment.

Compound/Drug	Cancer type	Cell line/animal model	Status	Effect	References
DOX	Colon cancer	CT26 cells	*In vitro*	Properly entered into tumors cells and maintained in the cells; Higher antitumor activity;	(104)
Vincristine	Brain cancer	MDA-MB-231 cells	*In vitro*	Longer residence time; Higher tissue concentrations; Improve the drug delivery to brain;	(105)
MTX and CUR	Breast cancer	MCF-7 cells	*In vitro*	Effectively deliver drug enhanced cytotoxic activity; Prolonged drug release	(106)
DOX and docosahexaenoic acid	Breast cancer	MCF-7 and 4T1 cells Balb/c nude mice	*In vitro* *In vivo*	Enhance water solubility; Improve pharmacokinetic profiles; More superior antitumor activity;	(111)
PTX	Oral carcinoma cell	KB cells	*In vitro*	Excellent targeting effects and greater anticancer effect;	(113)
MTO	Breast cancer	MCF-7 cells	*In vitro*	Enter into the resistant cancer cells by endocytosis; Efficiently enhanced cytotoxicity;	(118)

### 3.4 Alginates

#### 3.4.1 Drug targeted delivery

ALG-based nanosystems exhibited controlled drug release, increased stability, enhanced drug-loading capacity, and reduced immunogenicity, which renders them attractive biomaterials for cancer therapy applications (74, 75). Zhang et al. synthesized DOX-loaded glycyrrhetinic acid-modified ALG NPs (DOX/GA-ALG NPs) for liver-targeting drug delivery. The DOX/GA-ALG NPs resulted in a more potent anti-tumor effect in mice with H22 orthotopic liver tumors compared to other treatments, without causing any noticeable negative impact on normal liver tissue (136). A liposome was created in a recent study by using a conjugate of sodium ALG and cisplatin to specifically target EGFR-expressing tumors. Mice treated with CS-EGF-Lip exhibited significantly increased antitumor activity, enhanced delivery of cisplatin into ovarian tumor tissues and decreased nephrotoxicity compared to the other treatment groups (137).

Hydrogel-based systems have shown great potential for targeted anticancer drug delivery with high flexibility and controlled release behavior (67). Tolou et al. investigated a pressure-responsive nano gel based on ALG-Cyclodextrin for targeted delivery of 5-FU to HT-29 cells. Compared to free 5-FU, a higher intracellular accumulation of 5-FU and a significant extension of cell death through the apoptosis mechanism were observed. The data showed that the nanogel is cytocompatible, promising drug loading and controlled release (138).

#### 3.4.2 Drug aqueous solubility

ALG-based nanosystems have exhibited promising properties for encapsulating hydrophobic bioactive compounds. For instance, Dey and Sreenivasan have developed ALG-CUR conjugate for enhancing the solubility and stability of CUR (139). Sarika et al. developed a galactosylated ALG-CUR conjugate for improved delivery of CUR to hepatocytes. CUR can be targetedly delivered to hepatocytes, due to the asialoglycoprotein receptor on hepatocytes (140).

CUR has also shown potential in treating breast cancer (141). A magnetic ALG/CS NP loaded with CUR was developed for treating breast cancer. The NPs were designed to efficiently deliver CUR to both MDA-MB-231 breast cancer cells and HDF cells. Through manipulation of the number of CS and ALG layers on the NPs, the release of CUR can be controlled and sustained. The uptake efficiency of CUR was 3 to 6 times higher in MDA-MB-231 breast cancer cells treated with CUR-loaded NPs, as compared to those treated with free CUR (142).

#### 3.4.3 PH-sensitive capsules

ALG-based nanohybrids with suitable pH-responsive functions have been fabricated for sustained anticancer drug delivery. ALG, CS, and kappa-carrageenan (ALG/CS/KC) microcapsules are highly efficient in encapsulating 5-FU; and could be an effective and safe option for targeted delivery of anticancer drugs to the colon. Compared to ALG/CS and ALG, ALG/CS/KC releases the drug more slowly, and the polyelectrolyte complex shell of the microcapsules effectively prevents any sudden and explosive release of 5-FU (143). In another research, Justin et al. employed a water-in-oil emulsification technique for the synthesis of DOX-loaded ALG-CS therapeutic nanocarriers. DOX was encapsulated in the NP solution and could release steadily from the NP formulation in neutral pH and accelerated release in acidic pH (144).

#### 3.4.4 Drug-resistant

By combining silver NPs and TMX within an ALG core, the novel nanoformulation enables to effectively overcome endocrine resistance in breast cancer. The novel nanoformulation resulted in a 2.3-fold increase in reactive oxygen species induction, as well as a significant G2/M cell cycle arrest of 74.14% in MCF-7 cells. This multi-functional nanocomposite has the potential to be an effective targeted therapy for breast cancer through ROS-driven NF-κB pathway modulation (145). Some recent examples of ALG-based drug delivery and application are described in **Table 4**.

**Table 4:** A summary of Alginates‐based nanostructures for drug delivery in cancer treatment.

Compound/Drug	Cancer type	Cell line/animal model	Status	Effect	References
DOX	Liver cancer	H22 orthotopic liver tumors Kunming mice	*In vivo*	Enhance the concentration of DOX in the liver; Without apparent negative impact;	(119)
Cisplatin	Ovarian cancer	SKOV3 and A2780 cells Balb/c nude mice	*In vitro* *In vivo*	Inhibite cell growth and migration;decrease nephrotoxicity;	(120)
5-FU	Colon cancer	HT-29 cells	*In vitro*	Better cytocompatible; Higher 5-Fu intracellular accumulation and significant cell death extension;	(121)
CUR	Liver cancer	HepG2 cells	*In vitro*	Better and selective toward HepG2 cells; Enhanced anticancer activity;	(123)
CUR	Breast cancer	MDA-MB-231 cells	*In vitro*	Enhanced uptake efficiency and cytotoxicity to cancer cells;	(125)
DOX	Breast cancer	4T1 cells	*In vitro*	Entrap aqueous therapeutic payloads; Enhanced drug release in low pH range;	(127)
TMX	Breast cancer	MCF-7 cells	*In vitro*	Targeting and accumulation in tumors; Superior cytotoxic effect against breast cancer; Overcome NF-κB-based endocrine therapeutic resistance;	(128)

## 4 Other cancer drug delivery system

Except for glycan, nanocarriers are usually made from organic materials, such as lipids and proteins, or inorganic materials, such as graphene, gold, iron, and silicon-based (146–148). The use of proteins as structural material of nanocarriers offers several important advantages including non-toxic, good biocompatibility, good biodegradability, and High drug binding capacity (149–151). Protein NPs have been most extensively used for the delivery of anticancer drugs (152). Such as albumin, which can accumulate in solid tumors making it a potential carrier for targeted delivery of antitumor drugs (153). However, different molecular weights and immunogenicity are among the limitations of protein NPs and since proteins are often hydrophilic molecules, their NPs swell by absorbing water and the drug spreads rapidly outside (150). Besides, proteins will cover the surface of the nanocarriers to form a protein crown which could alter protein NP size and surface properties, making their arrival at target sites difficult (146, 154). Because of high stability, low degradability, and *in vivo* compatibility, lipid NPs have been widely employed for drug delivery (138–140). Solid-lipid NPs emerged as a dominant lipid-based nanocarrier in diverse drug delivery systems (140). However, the main disadvantages associated with Solid-Lipid NPs are low drug loading ability and drug ejection while preservation, etc (155, 156).

Inorganic NPs have unique physical, electrical, magnetic, and optical properties, which are not possible with traditional lipid or polymer-based NPs (157, 158). For example, magnetic-based systems utilizing superparamagnetic NPs and various configurations of magnetic fields and field gradients make magnetic targeting a viable clinical treatment modality (159, 160). However, inorganic nanocarriers are not biodegradable and cannot deliver high-potency drugs, so they must be combined with organic materials (160, 161). They are also limited in their clinical application by low solubility and toxicity concerns, especially in formulations using heavy metals (162, 163).

4. Glycan and glycoconjugates as drug carriers in application of cancer immunotherapy

Tumor immunotherapy has garnered considerable attention in recent years, aiming to modulate the tumor immune microenvironment (TME), activate the immune system for tumor cell eradication, and instigate immune surveillance (164). Immune checkpoint inhibitors, such as antibodies targeting cytotoxic T-lymphocyte-associated protein 4 (CTLA-4), programmed death-1 (PD-1), and programmed death-ligand 1 (PD-L1), have markedly improved cancer patient outcomes, but evoke several adverse effects, which often limit their clinical use (165, 166). Transdermal drug delivery offers advantages, including bypassing the gastrointestinal tract, mitigating gastrointestinal irritation and the liver first-pass effect, directly reaching the lesion site, and reducing unnecessary adverse reactions. Utilizing fluorocarbon-modified CS, non-invasive transdermal delivery of immune checkpoint blockade antibodies effectively inhibits melanoma growth and diminishes systemic toxicity (165).

The augmentation of immunotherapy through targeted inhibition of immune suppression or activation of immune cells is increasingly gaining traction (167). Immunosuppressive tumour immune microenvironments (TME) limit the success of immune checkpoint blockade. Zhang et al. have developed chitosan-based nano-micelles that accurately and effectively activate the Cyclic GMP-AMP synthase-stimulator of interferon gene signaling pathway, thereby remodeling the TME and effectively inhibiting tumor growth (164). Immunoregulation mediated by tumor-associated myeloid cells (TAMCs) represents a major hurdle in cancer immunotherapy. TAMCs, including tumor-associated macrophages and myeloid-derived suppressor cells, constitute the primary immune-suppressive component of TME, secreting various immune-regulatory factors such as IL-6 and TGF-β (168–170). However, hyaluronic acid-bilirubin nanoparticles have demonstrated the ability to convert TAMCs into a less immunosuppressive phenotype and induce immunogenic cell death in tumor cells, resulting in potent anti-tumor effects and synergizing strongly with immune checkpoint blockade therapy.

Furthermore, the buildup of extracellular adenosine triggers the A2A receptor cascade, promoting cancer progression by fostering immunosuppressive cellular responses (171–173).A2A receptor antagonists hold promise for treating cancers by enhancing immunotherapy when combined with other therapeutic agents. Hamed et al. employ chitosan nanoparticles as carriers to deliver A2A receptor antagonists, targeting the overexpression of adenosine receptors in cancer cells. This strategy involves combining A2A receptor antagonists with methotrexate to bolster the immune system, alleviate immune suppression, and enhance the recognition of cancer cells for improved therapeutic outcomes (174).

## 5 Conclusions

Glycans, which are complex carbohydrates found on the surface of cells and proteins, have been shown to play an important role in targeted drug delivery for cancer treatment and be used to improve the pharmacokinetics and pharmacodynamics of drugs. As a natural carrier, glycan can protect drugs from degradation and clearance by the body’s immune system. By modifying drugs with glycans, it is possible to extend their half-life in the body and enhance their bioavailability. In addition, glycans have high biocompatibility and biodegradability, making them an attractive option for drug delivery *in vivo*. Drugs can be conjugated with glycan, allowing them to be targetedly delivered to cancer cells. One of the key advantages of using glycans as a drug delivery system is their ability to selectively bind to specific cellular receptors that are overexpressed on the surface of cancer cells. This targeted delivery approach minimizes off-target effects and reduces toxicity to healthy tissues. Furthermore, glycans can be engineered to carry a wide range of payloads, such as chemotherapeutic drugs, antibodies, and nucleic acids, which can be released in a controlled manner at the site of the tumor. Glycan-based drug delivery systems can also improve the pharmacokinetics and pharmacodynamics of the cancer treatment drug, leading to better efficacy and reduced toxicity. Overall Glycans have shown promising potential as drug delivery platforms for cancer therapy. However, the complex structure of glycans can make their synthesis and modification challenging and time-consuming, which can limit the scalability and reproducibility of glycan-based scaffold production. Glycans are susceptible to degradation by enzymes and other factors in the body, which can limit their durability and effectiveness as a scaffold material. Furthermore, it is crucial to thoroughly assess the destiny of NPs and their biodegradability, taking into account nanotoxicology concerns. In addition, some of the studies conducted on the efficacy of treatments are preclinical trials that use cultured cells or animal models. Therefore, it is crucial to confirm these findings through clinical trials involving patients to establish their effectiveness and safety in a clinical setting.

Glycan-based drug delivery systems are still in the early stages of development, but ongoing research efforts have shown promising results. With further optimization and refinement of the technology, glycans have the potential to become a valuable tool for cancer therapy, providing a safe and effective means for targeted drug delivery to cancer cells.

## Author contributions

HQ: Writing – original draft, Writing – review & editing. YT: Writing – original draft, Writing – review & editing. RD: Methodology, Supervision, Writing – original draft. AW: Funding acquisition, Writing – review & editing. JL: Funding acquisition, Investigation, Writing – review & editing, Writing – original draft.
